# Supplementary material for: Biogeochemistry of Dominant Plants and Soils in Shewushan Gold Lateritic Deposit, China
Source: Plants (Basel). 2021 Dec 23;11(1):38. doi: 10.3390/plants11010038 (PMC8747375; doi:10.3390/plants11010038)
Supplement: Supplementary file 1 [file plants-11-00038-s001.zip › ME-MS61(EN).pdf]

ME-MS61
Ultra-Trace Level Method Using ICP-MS and ICP-AES

Sample Decomposition:

HF-HNO_3-$HClO_4$ acid digestion, HCl leach

Analytical Method:

Inductively Coupled Plasma - Atomic Emission Spectroscopy (ICP - AES)
Inductively Coupled Plasma - Mass Spectrometry (ICP-MS)

A prepared sample is digested with perchloric, nitric and hydrofluoric acids. The residue is leached with dilute hydrochloric acid and diluted to volume. It is then analyzed by inductively coupled plasma-atomic emission spectrometry and inductively coupled plasma-mass spectrometry. Results are corrected for spectral interelement interferences.

NOTE: Four acid digestions are able to dissolve most minerals; however, although the term "*near-total*" is used, depending on the sample matrix, not all elements are quantitatively extracted.

Element	Symbol	Units	Lower Limit	Upper Limit
Silver	Ag	µg/g	0.01	100
Aluminum	Al	%	0.01	50
Arsenic	As	µg/g	0.2	9,900
Barium	Ba	µg/g	10	10,000
Beryllium	Be	µg/g	0.05	990
Bismuth	Bi	µg/g	0.01	9,900
Calcium	Ca	%	0.01	50
Cadmium	Cd	µg/g	0.02	990
Cerium	Ce	µg/g	0.01	500
Cobalt	Co	µg/g	0.1	9,900
Chromium	Cr	µg/g	1	10,000
Cesium	Cs	µg/g	0.05	500
Copper	Cu	µg/g	0.2	10,000
Iron	Fe	%	0.01	50
Gallium	Ga	µg/g	0.05	9,900
Germanium	Ge	µg/g	0.05	500

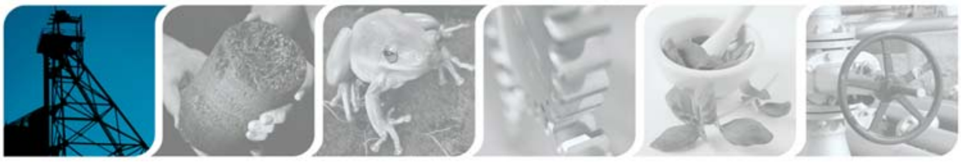

Element	Symbol	Units	Lower Limit	Upper Limit
Hafnium	Hf	µg/g	0.1	500
Indium	In	µg/g	0.005	500
Potassium	K	%	0.01	10
Lanthanum	La	µg/g	0.5	5,000
Lithium	Li	µg/g	0.2	1,000
Magnesium	Mg	%	0.01	50
Manganese	Mn	µg/g	5	100,000
Molybdenum	Mo	µg/g	0.05	9,900
Sodium	Na	%	0.01	10
Niobium	Nb	µg/g	0.1	500
Nickel	Ni	µg/g	0.2	9,900
Phosphorous	P	µg/g	10	10,000
Lead	Pb	µg/g	0.5	9,900
Rubidium	Rb	µg/g	0.1	9,900
Rhenium	Re	µg/g	0.002	50
Sulphur	S	%	0.01	10
Antimony	Sb	µg/g	0.05	10,000
Scandium	Sc	µg/g	0.1	9,900
Selenium	Se	µg/g	1	1,000
Tin	Sn	µg/g	0.2	500
Strontium	Sr	µg/g	0.2	10,000
Tantalum	Ta	µg/g	0.05	100
Tellurium	Te	µg/g	0.05	500
Thorium	Th	µg/g	0.2	5,000
Titanium	Ti	%	0.005	10
Thallium	Tl	µg/g	0.02	1,000
Uranium	U	µg/g	0.1	9,900
Vanadium	V	µg/g	1	10,000
Tungsten	W	µg/g	0.1	1,000
Yttrium	Y	µg/g	0.1	500
Zinc	Zn	µg/g	2	9,900
Zirconium	Zr	µg/g	0.5	500
