# Supplementary material for: Biogeochemistry of Dominant Plants and Soils in Shewushan Gold Lateritic Deposit, China
Source: Plants (Basel). 2021 Dec 23;11(1):38. doi: 10.3390/plants11010038 (PMC8747375; doi:10.3390/plants11010038)
Supplement: Supplementary file 1 [file plants-11-00038-s001.zip › ME-VEG41(EN).pdf]

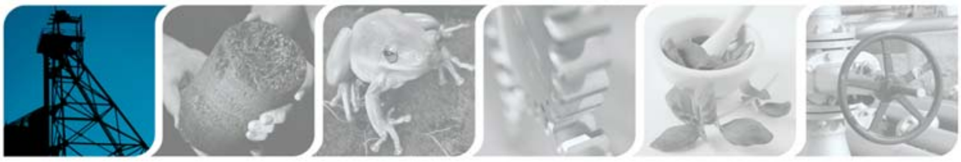

ME-VEG41
Vegetation: Ultra-Trace Level Using ICP-MS and ICP-AES

Sample Decomposition:

Nitric acid/Hydrochloric acid

Analytical Method:

Inductively Coupled Plasma-Atomic Emission Spectroscopy (ICP-AES)
Inductively Coupled Plasma - Mass Spectrometry (ICP-MS)

Plants may be viewed as large-scale geochemical sampling devices, with root systems that can selectively absorb elements from a large volume of soil, groundwater, and even bedrock. In any biogeochemistry program careful selection of plant species and organ of interest is important as biological effects control geochemical response.

The Vegetation Super Trace methods are analyzed via ICP-MS instrumentation utilizing collision/reaction cell technologies to provide the lowest detection limits available. The instrument has been optimized for long-term robust ICP-MS signal stability, in particular for samples with high Ca content.

A prepped sample is cold digested with nitric acid for 8 hours before being transferred to hot block for 15 minutes at 85°C followed by 2 hours at 115°C. The samples are subsequently cooled and brought up to volume with HCl. The resulting solution is mixed thoroughly and analyzed by ICP-MS and ICP-AES corrected for spectral interferences.

Analyte	Symbol	Units	Lower Limit	Upper Limit
Gold	Au	µg/g	0.0002	100
Silver	Ag	µg/g	0.001	100
Aluminum	Al	%	0.01	25
Arsenic	As	µg/g	0.01	10,000
Boron	B	µg/g	1	10,000
Barium	Ba	µg/g	0.1	10,000
Beryllium	Be	µg/g	0.01	1,000
Bismuth	Bi	µg/g	0.001	10,000
Calcium	Ca	%	0.01	40
Cadmium	Cd	µg/g	0.001	2,000
Cerium	Ce	µg/g	0.003	500
Cobalt	Co	µg/g	0.002	10,000

Analyte	Symbol	Units	Lower Limit	Upper Limit
Chromium	Cr	µg/g	0.01	250
Cesium	Cs	µg/g	0.005	500
Copper	Cu	µg/g	0.01	10,000
Iron	Fe	µg/g	1	50,000
Gallium	Ga	µg/g	0.004	10,000
Germanium	Ge	µg/g	0.005	500
Hafnium	Hf	µg/g	0.002	500
Mercury	Hg	µg/g	0.001	100
Indium,	In	µg/g	0.005	500
Potassium	K	%	0.01	10
Lanthanum	La	µg/g	0.002	10,000
Lithium	Li	µg/g	0.1	10,000
Magnesium	Mg	%	0.001	30
Manganese	Mn	µg/g	0.1	50,000
Molybdenum	Mo	µg/g	0.01	10,000
Sodium	Na	%	0.001	10
Niobium	Nb	µg/g	0.002	500
Nickel	Ni	µg/g	0.04	10,000
Phosphorus	P	%	0.001	5
Lead	Pb	µg/g	0.01	10,000
Palladium	Pd	µg/g	0.001	100
Platinum	Pt	µg/g	0.001	100
Rubidium	Rb	µg/g	0.01	10,000
Rhenium	Re	µg/g	0.001	50
Sulphur	S	%	0.01	10
Antimony	Sb	µg/g	0.01	10,000
Scandium	Sc	µg/g	0.01	10,000
Selenium	Se	µg/g	0.005	1,000
Tin	Sn	µg/g	0.01	500
Strontium	Sr	µg/g	0.02	10,000
Tantalum	Ta	µg/g	0.001	500
Tellurium	Te	µg/g	0.02	500
Thorium	Th	µg/g	0.002	10,000
Titanium	Ti	%	0.001	10
Thallium	Tl	µg/g	0.002	10,000
Uranium	U	µg/g	0.005	10,000
Vanadium	V	µg/g	0.05	10,000
Tungsten	W	µg/g	0.01	10,000
Yttrium	Y	µg/g	0.003	500

Analyte	Symbol	Units	Lower Limit	Upper Limit
Zinc	Zn	µg/g	0.1	10,000
Zirconium	Zr	µg/g	0.02	500
