# Supplementary figures and images for: Biogeochemistry of Dominant Plants and Soils in Shewushan Gold Lateritic Deposit, China
Source: Plants (Basel). 2021 Dec 23;11(1):38. doi: 10.3390/plants11010038 (PMC8747375; doi:10.3390/plants11010038)

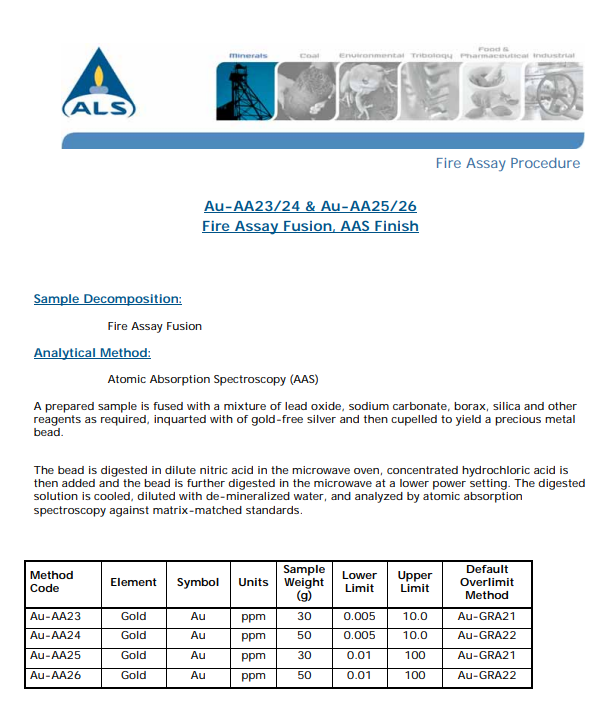

Supplement: Supplementary file 1 [file plants-11-00038-s001.zip › Au-AA23.docx]

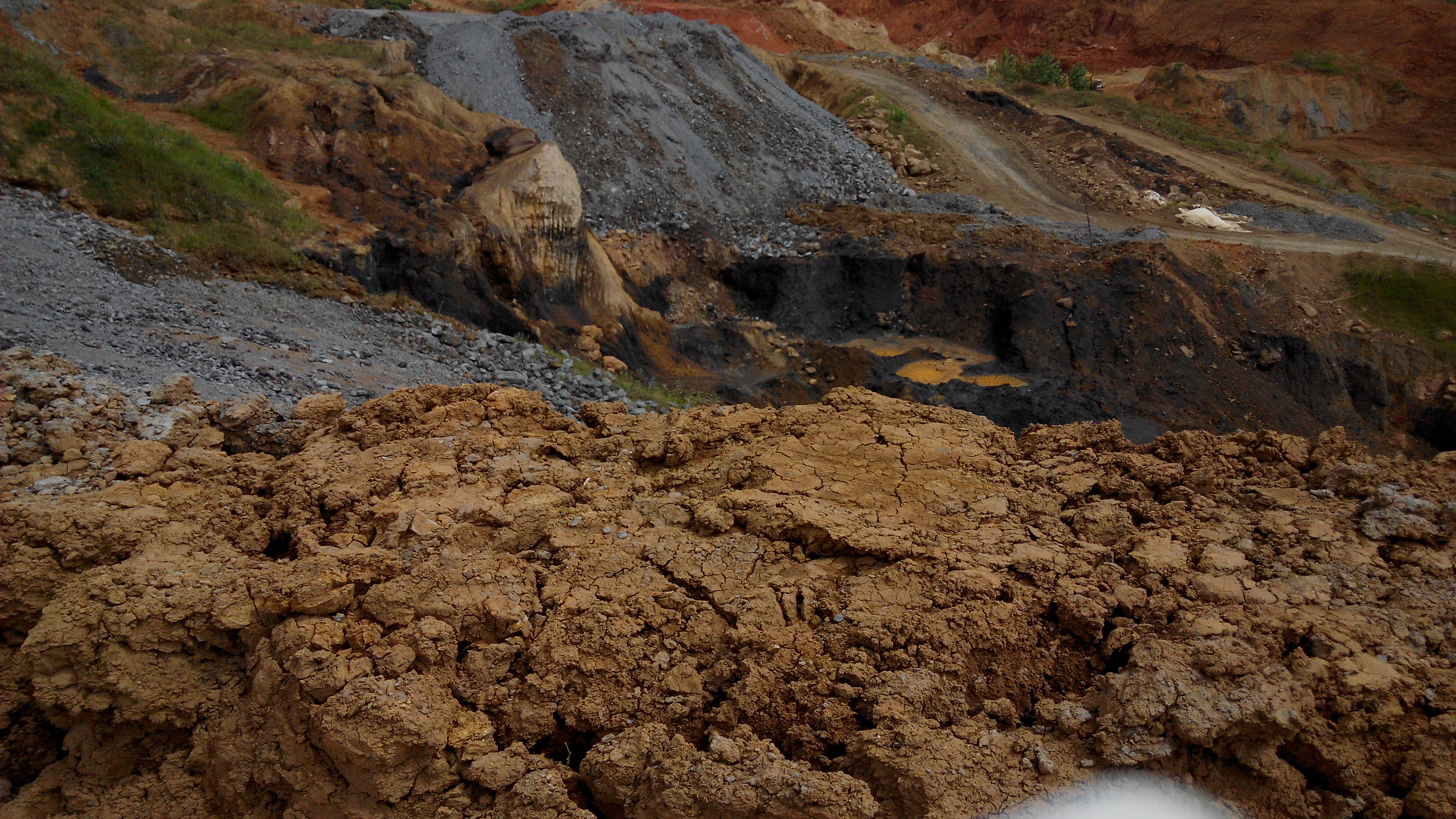

Supplement: Supplementary file 1 [file plants-11-00038-s001.zip › IMG_20140624_140906.jpg]

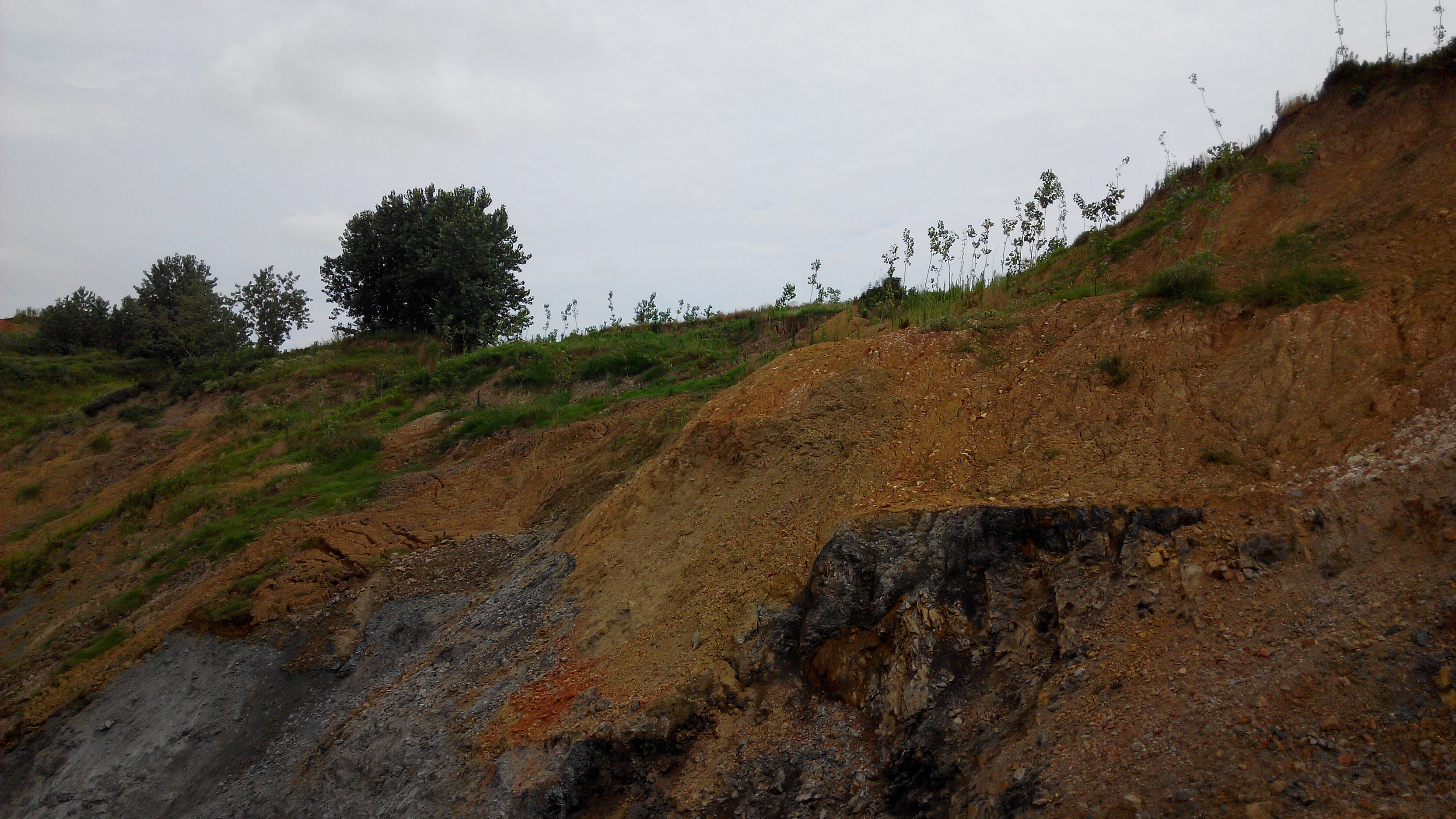

Supplement: Supplementary file 1 [file plants-11-00038-s001.zip › IMG_20140624_142221.jpg]

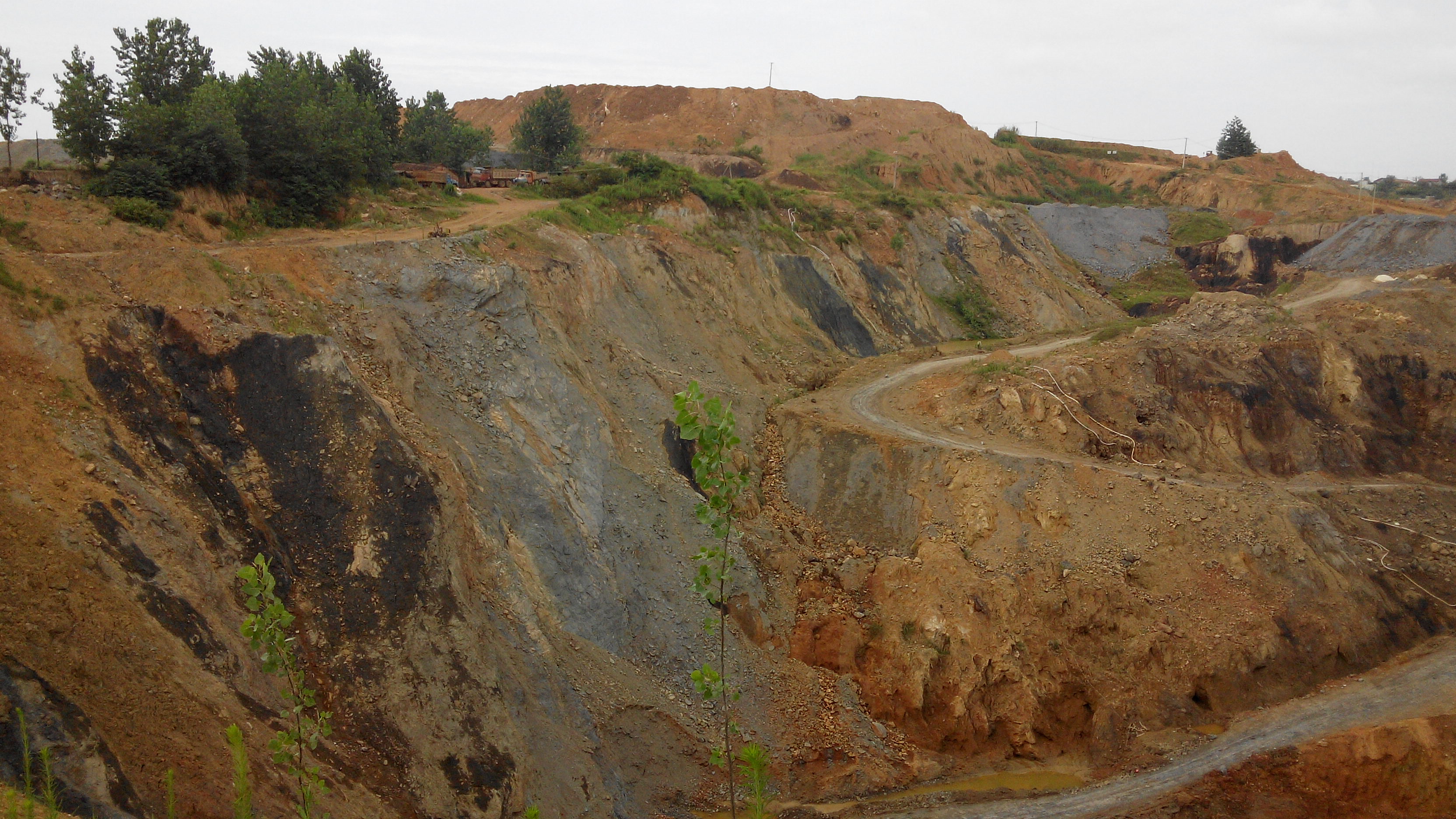

Supplement: Supplementary file 1 [file plants-11-00038-s001.zip › IMG_20140624_153211.jpg]

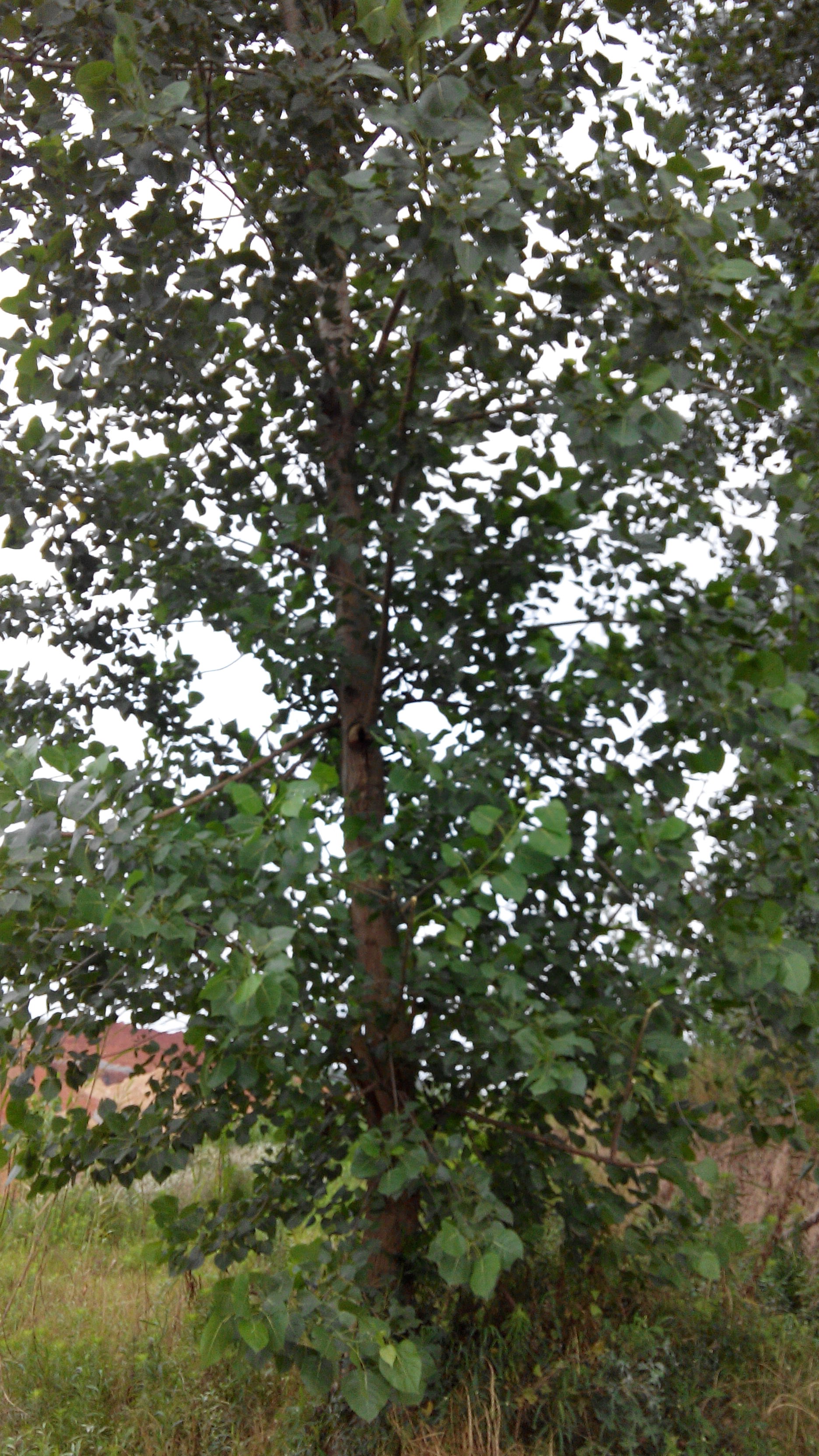

Supplement: Supplementary file 1 [file plants-11-00038-s001.zip › IMG_20140624_153957.jpg]

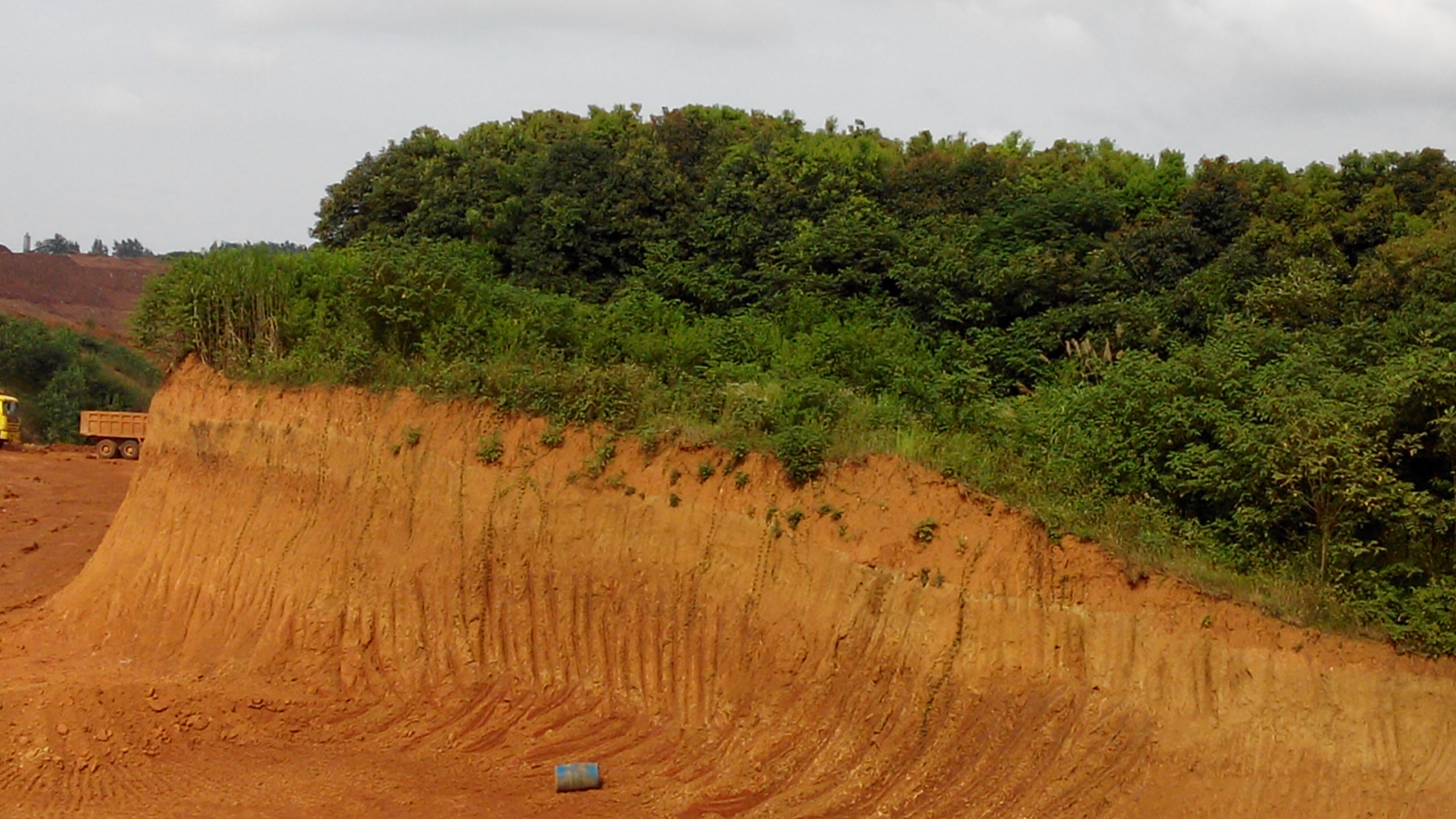

Supplement: Supplementary file 1 [file plants-11-00038-s001.zip › IMG_20140624_160751.jpg]
